# The Efficacy and Safety of Combined Senna and Probiotic-Based Bowel Preparation for Colonoscopy in Children

**DOI:** 10.7759/cureus.10180

**Published:** 2020-09-01

**Authors:** Dhanasekhar Kesavelu

**Affiliations:** 1 Paediatric Gastroenterology, Apollo Children's Hospital, Chennai, IND

**Keywords:** pediatrics, sennoside, tolerability, bowel preparation, india, bacillus coagulans snz1969

## Abstract

Background and objective

Bowel preparation (BP) is a major determinant of accurate and successful colonoscopy and its outcomes in children and adults. The present study aimed to evaluate the effectiveness and safety of a senna alkaloid and probiotic-based BP in children undergoing elective colonoscopy.

Methods

Children aged 2-15 years who underwent esophagogastroduodenoscopy (OGD) and colonoscopy for various indications from December 2018 to May 2019 at the Apollo Children’s Hospital who were prescribed a senna and probiotic-based [*Bacillus coagulans (B. coagulans)*]* *preparation before the procedure were included in the study. The effectiveness of the bowel preparation was assessed using the Boston Bowel Preparation Scale (BBPS). Safety assessment was performed by recording adverse events in the study.

Results

Successful bowel preparation was observed in all patients according to BBPS, and the mean BBPS score was 2.93 ±0.25, indicating good bowel clearance. About 29 (96.67%) patients showed accessible terminal ileum. There were no major side effects reported in the study. The formulation was found to be palatable and acceptable by 100% of patients.

Conclusion

The study revealed the administration of the novel BP to be effective and safe in children undergoing elective colonoscopy for various indications.

## Introduction

Colonoscopy is essential for the diagnosis and management of various gastrointestinal (GI) tract disorders and is presently the standard investigation for assessing the mucosa of the entire colon. Children usually undergo the procedure for diagnostic purposes for indications such as GI bleeding, abdominal pain, and diarrhea, and/or any other indication based on the discretion of the clinician [1]. Hence, the procedure should be accurate and safe. The accuracy and safety of the procedure are highly dependent on colon cleansing, i.e., bowel preparation (BP).

Evidence has established that insufficient bowel cleansing cannot detect neoplastic lesions and can result in a higher procedure cancellation rate, which in turn is liable to increase total healthcare expenditure and make the procedure lengthier, leading to a higher risk of complications and inconvenience and a high level of emotional trauma to the child and the family [2,3]. An ideal BP agent should be effective, safe, tolerable, and should have a high compliance rate [4].

Commonly used agents for BP include iso-osmotic agents: high-volume and low-volume polyethylene glycol (PEG) preparations, hypo-osmotic agents, hyperosmotic agents, sodium sulfate, magnesium citrate, and sodium phosphate; and laxatives [2]. Although a variety of bowel preparations are available for pediatric colonoscopies at present, none of the preparations meet all the requirements for an ideal BP agent. PEG agents are the most commonly used BP agents, and several studies have assessed their safety and efficacy in the pediatric population [2,5]. Although PEG agents (with or without electrolytes) have been shown to be effective and well-tolerated, a survey conducted by the North American Society for Pediatric Gastroenterology, Hepatology, and Nutrition (NASPGHAN) among the pediatric population reported that PEG agents needed ‘too much volume’ to drink and led to ‘a lengthy procedure,’ resulting in cancellation or rescheduling of the procedure in 20% of cases [5]. However, apart from the studies on PEG, very little evidence is available regarding the efficacy and tolerability of various other BP agents used in the pediatric population. Additionally, indications on the duration of liquid diet and the need for stimulant adjunctive therapy (including laxatives, flavoring) are found to be inconsistent among conducted studies. In this regard, there is a need gap for developing and evaluating an effective and palatable BP agent for the pediatric population undergoing elective colonoscopy [5].

The BP agent M Sip Lax® straws (Inzpera Healthsciences Ltd, Mumbai, India) used in our study has two active ingredients: stimulant laxative (senna) and a probiotic [*Bacillus coagulans (B. coagulans) ​​​​*SNZ1969]. *B. coagulans*, a lactic acid, spore-forming bacterial species of the genus *Bacillus* has an optimum growth temperature of 50 °C and can tolerate temperatures in the range of 30 °C-55 °C. Additionally, *B. coagulans* enhances the microbial flora in the gastrointestinal tract and initiates immunostimulation [6]. Furthermore, since the spores of *B. coagulans* are acid-resistant, almost all the spores reach the intestine, and the lactic acid increases the fecal moisture content, thereby conferring beneficial effects, especially with regard to the alleviation of constipation. In a study involving healthy individuals with symptoms of constipation, treatment with *B. coagulans* SANK 70258 [n=20, 1 x 108 colony-forming units (CFUs)/day, two weeks] resulted in an improvement in scores with respect to fecal shape, color, odor, pH, and defecation frequency compared to the scores reported before ingestion [7]. In another study, *B. coagulans* lilac-01 (n=138, 1 x 108 CFUs/day, two weeks) resulted in a significant improvement in the fecal size, color, odor, straining, the sensation of incomplete evacuation, and defecation frequency in individuals with functional constipation [8].

Senna, commonly used as a laxative, is an established product worldwide and is recommended by the National Institute for Health and Care Excellence (NICE), UK for the management of constipation in children and young adults [9].

*B. coagulans* and senna present in this combination product have two different mechanisms to ensure good bowel clearance. *B. coagulans, *after arriving in the stomach in its spore form, starts absorbing water, swells, and begins the germination process. Upon reaching the duodenum, spores germinate within four to six hours and multiply rapidly with approximately 85% of their starting material reaching the intestinal tract. After germination, *B. coagulans* becomes metabolically active in the intestine and produces levorotatory lactic acid, which is easily metabolized during glycogen synthesis. Eventually, *B. coagulans* is excreted slowly via feces for around seven days after discontinuation of ingestion, as it is a transient colonizing probiotic. Senna is hydrophilic in nature and is not readily absorbed by the gut. However, once senna reaches the intestine, it is deconjugated and reduced to the active form by indigenous microflora. It is cleaved further to active metabolites by the bacteria. These active metabolites are poorly absorbed systemically but evoke secretion and metabolic changes in the colon [10].

Our study primarily aims to add to the current evidence for the effectiveness and safety of this first-of-its-kind combination, M Sip Lax® straws consisting of a probiotic (*B. coagulans* SNZ1969) and senna alkaloid for clearing the bowel in Indian pediatric patients with various gastrointestinal complaints that require a colonoscopy.

## Materials and methods

Study design

The study was a prospective, single-center, non-randomized, observational study. Efficacy and safety data were collected simultaneously.

Study participants

Children aged 2-15 years who underwent esophagogastroduodenoscopy (OGD) and colonoscopy or colonoscopy alone for various indications between December 2018 and May 2019 at Apollo Children’s Hospital, Chennai and who were prescribed this BP were included in the study. The age, gender, and indications for colonoscopy for all patients were recorded.

Informed consent and ethical approval

The study was approved by the Institutional Ethics Committee and was compliant with the Ethical Guidelines for Biomedical Research on Human Subjects by the Indian Council of Medical Research (ICMR), Govt. of India. Informed consent was obtained from all individual participants included in the study.

Study interventions

The patients received the product M Sip Lax®, containing one billion CFUs of *B. coagulans* SNZ1969 [the accession number for 16S ribosomal RNA (rRNA) is KC146407] originally isolated from green malt, and 7.5 mg of sennoside. The product was taken orally in a glass of water before the colonoscopy procedure, per the physician’s discretion [10,11]. The dose was prescribed by the physician, based on the patient’s age (Table 1).

Age of children

The recommended number of M Sip Lax® straws for bowel preparation based on the children's age is presented in Table 1.

**Table 1:** Recommended number of M Sip Lax® straws according to the patients' age for bowel preparation

Age of children	Recommended number of M Sip Lax® straws
1 month–4 years	2 straws
4–6 years	3 straws
6+ years	4 straws

Study procedure

**Figure 1:**
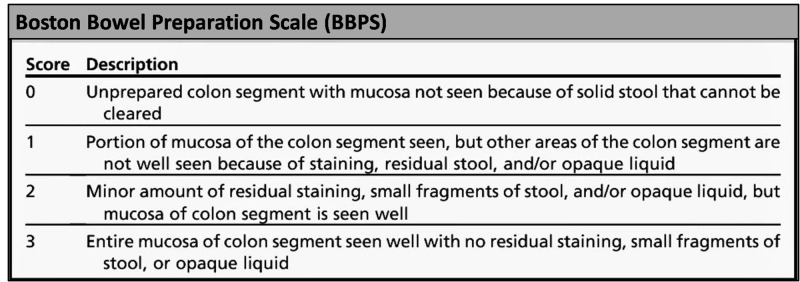
The Boston Bowel Preparation Scale

A liquid diet restriction was imposed on the patients before the procedure for a minimum period of 12 hours. All patients received rectal enema on the morning before the procedure. The colonoscopy procedure was performed by an endoscopist/gastroenterologist with 18 years of experience under general anesthesia with anesthetist supervision. The effectiveness of BP to cleanse the bowel was assessed using the standardized Boston Bowel Preparation Scale (BBPS). The four-point scoring system of BBPS is shown in Figure 1 [12].

Safety and tolerability

Safety and tolerability were evaluated based on compliance and a BBPS score of worse than 3.

Data analysis

Statistical analysis was performed using Microsoft Excel (Microsoft, Redmond, WA). Values were expressed as mean ± standard deviation.

## Results

Demographics and baseline characteristics

In total, data of 30 patients were included in the study. The mean age of the patients was 9.47 ±3.87 years; 18 patients were male and 12 were female (60:40). Various indications for colonoscopy in pediatric patients included inflammatory bowel disease (IBD) (Crohn’s disease, ulcerative colitis, indeterminate colitis, bleeding per rectum, recurrent diarrhea, etc.). The majority of patients (n=5) were advised follow-up colonoscopy for Crohn’s disease, followed by non-infective colitis (n=4), and chronic abdominal pain (n=4) (Table 2).

**Table 2:** Demographic data and indications for colonoscopy SD: standard deviation

Patient characteristics	Value
Age in years, mean ±SD	9.47 ±3.87
Gender, n (%)	Male: 18 (60). Female: 12 (40)
Indications for colonoscopy: abdominal pain, bleeding per rectum, chronic abdominal pain, chronic diarrhea, Crohn’s disease, suspected food allergy, inflammatory bowel disease (IBD), mesenteric lymphadenopathy, non-infective colitis, mucus per rectum, non-infective colitis, recurrent diarrhea, ulcerative colitis; n (%)	1 (3.33), 3 (10), 4 (13.33), 1 (3.33), 5 (16.67), 1 (3.33), 4 (13.33), 1 (3.33), 1 (3.33), 1 (3.33), 4 (13.33), 2 (6.67), 1 (3.33)

Efficacy of BP

The dosing frequency of the product was decided based on the patients' age (Table 1). Two (6.67%) patients received two doses, six (20%) received three doses, and 22 (73.33%) patients received four doses of the product.

**Figure 2:**
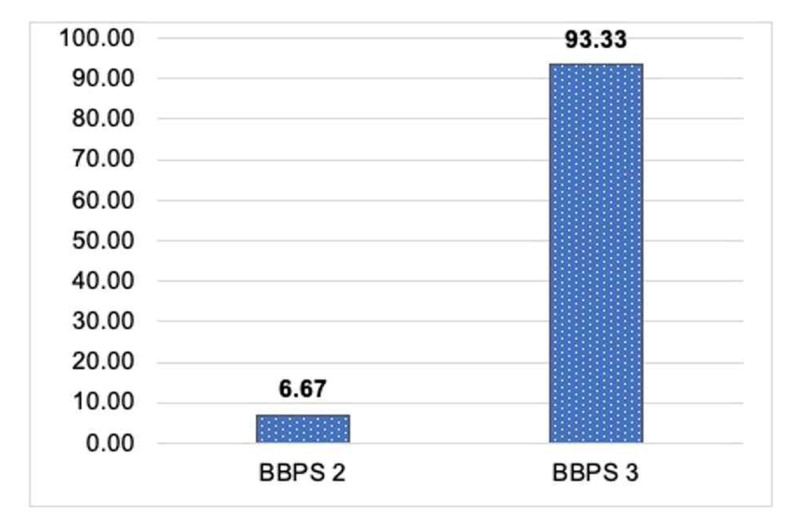
BBPS for all patients BBPS: Boston Bowel Preparation Scale

Successful BP was observed in all patients according to BBPS, and the mean BBPS score was 2.93 ±0.25, indicating good bowel clearance (Figure 2). Out of 30 patients, two (6.67%) had a BBPS of 2, while 28 (93.33%) had a BBPS of 3, indicating good bowel clearance. About 29 (96.67%) patients had accessible terminal ileum.

Safety and tolerability

The treatment was well-tolerated, and non-compliance with the medication among patients was rarely noted.

## Discussion

In this study, we showed that the BP agent consisting of a probiotic (*B. coagulans* SNZ1969) and senna alkaloid-based formulation was safe and effective in pediatric patients in our cohort who underwent OGD and colonoscopy. We evaluated this unique combination in view of the benefits its active components can offer during BP. In this regard, clinical studies have reported improved bowel movement and fecal properties after the administration of *B. coagulans* in the adult population [7,8,13]. It has been observed that disturbances in the composition and stability of the gut microbiota affect motility and secretory functions [13]. Hence, the addition of probiotics is necessary to avoid gut dysbiosis in children with gastrointestinal disease. Radaelli et al. have reported that senna preparation was better tolerated than high-volume PEG solution and exhibited fewer side effects compared to sodium phosphate (NaP) solution in a colon-cleansing study. Although they reported abdominal pain as a common complaint among patients who received senna, it did not affect the efficacy of senna for BP [14,15]. Another study by Yenidogan et al. reported that same-day administration of senna was safe and effective for bowel cleansing before colonoscopy and that patients were positive about using the same product in the future [16]. Another study conducted in the pediatric population showed that a three-day regimen of senna was comparable to a one-day regimen of PEG-bisacodyl [17]. A clinical trial conducted by Santos-Jasso et al. compared the effects of senna vs. PEG in constipated pediatric patients with anorectal malformation; the trial suggested that senna should be the laxative of choice for bowel management in pediatric patients [18]. In line with the previous literature, our unique combination of senna and *B. coagulans* thus not only addresses gut dysbiosis but also increases peristaltic movement by promoting healthy bowel movement. It also alleviates the adverse effects of constipation [10].

In addition to their efficacy, other advantages of BP agents are related to ingestion volume, taste, and tolerability. The main disadvantages of PEG agents include the large volume (4 L) that patients need to ingest and the unpleasant, salty taste of sodium sulfate. A randomized clinical trial comparing the efficacy of a lower volume (2 L) vs. conventional dose (4 L) did not show any difference in terms of the cleanliness of the entire colon. However, cleanliness in the right colon, which is an important aspect for screening, was not satisfactory with the smaller-volume dose [19]. In this aspect, the major advantage of this combination [10] is that the volume that needs to be ingested is much lower compared to PEG agents. This combination is packed in a straw that has a filter at both ends and beads that contain active ingredients. The straw is placed in a glass of water and the entire content of the straw and glass should be emptied at bedtime (for routine/maintenance use). In short, the patient consumes only a glass of water (normally 180-200 mL). The combination has a banana flavor, which is palatable to pediatric patients. The flavoring may improve compliance.

The quality of the diet affects the quality of cleansing. Studies have found that liquid-only diets result in better bowel cleansing [4]. However, a liquid-only diet is associated with nausea, headache, vomiting, and higher rates of procedure cancellation. In this regard, more well-defined studies are needed to understand the role of diet in achieving adequate cleansing. Our product does not need any specific diet restriction (except for the adherence to a liquid diet the day before the colonoscopy). This is possibly why we did not observe any side effects associated with a restricted diet.

As observed during our study, the efficacy of bowel cleanliness was satisfactory, with a mean BBPS score of 2.93. We chose the BBPS scale to rate the quality of bowel preparation; the scale avoids inter-observer variability in rating but indicates various degrees of bowel cleanliness [12]. This preparation was palatable, and the protocol was acceptable to the study participants. As the volume ingested was much lesser than the conventional volume, better compliance and a lower rate of cancellation could be achieved. Also, we did not observe the BP agent failing in any child. Hence, we can consider this preparation safe and tolerable for BP in pediatric patients.

Our study has some limitations. We have not performed a cost-effective analysis of this preparation. In this regard, factors such as the cost of repeat colonoscopy and the cost of senna and probiotic with respect to widely used PEG solutions need to be considered. We are aware that BBPS does not apply to pediatric patients; however, we have made an attempt to translate the BBPS to pediatric patients. Hence, we did not perform a segment-wise analysis of bowel clearance. Future studies are warranted to evaluate the efficacy of this novel combination in a larger pediatric population. Our study also highlights the beneficial effect of senna and probiotic-based BP for geriatric patients who may need frequent colonoscopies or for patients who may need frequent colonoscopies for any other indication or may be unwilling to take a large amount of fluids before colonoscopy; it may also be beneficial for colonic cancer surveillance/screening.

## Conclusions

This novel combination for bowel cleansing is completely different from the existing preparations. Our study presented evidence for the effectiveness and safety of senna and probiotic-based combination BP for children undergoing elective colonoscopy for various indications. The results indicate that this senna and probiotic-based BP can serve as a promising, viable option for the pediatric population undergoing elective colonoscopy. We sincerely believe that this study not only adds to the current evidence for the importance of efficient bowel cleaning in the pediatric population but also provides a viable BP option for practicing clinicians in the country.
